# Case for diagnosis. Ichthyosiform mycosis fungoides^☆^

**DOI:** 10.1016/j.abd.2021.10.008

**Published:** 2022-09-13

**Authors:** Luciana Baptista Pereira, Natália de Paiva Sobreira, Vanessa Barreto Rocha

**Affiliations:** Dermatology Service, Faculty of Medicine, Hospital das Clínicas, Universidade Federal de Minas Gerais, Belo Horizonte, MG, Brazil

## Case report

**Figure 1:**
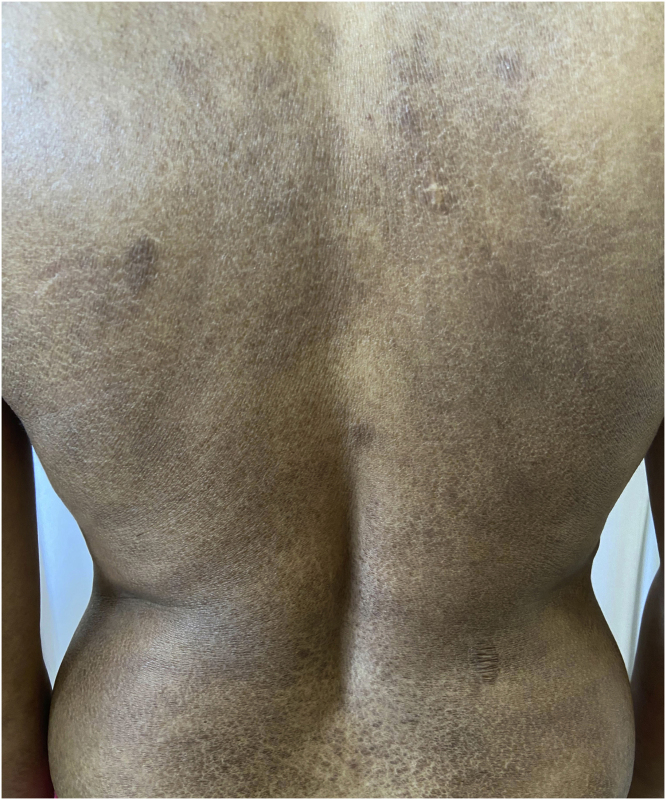
Hyperchromic plaques with ichthyosiform desquamation on the posterior trunk region.

**Figure 2:**
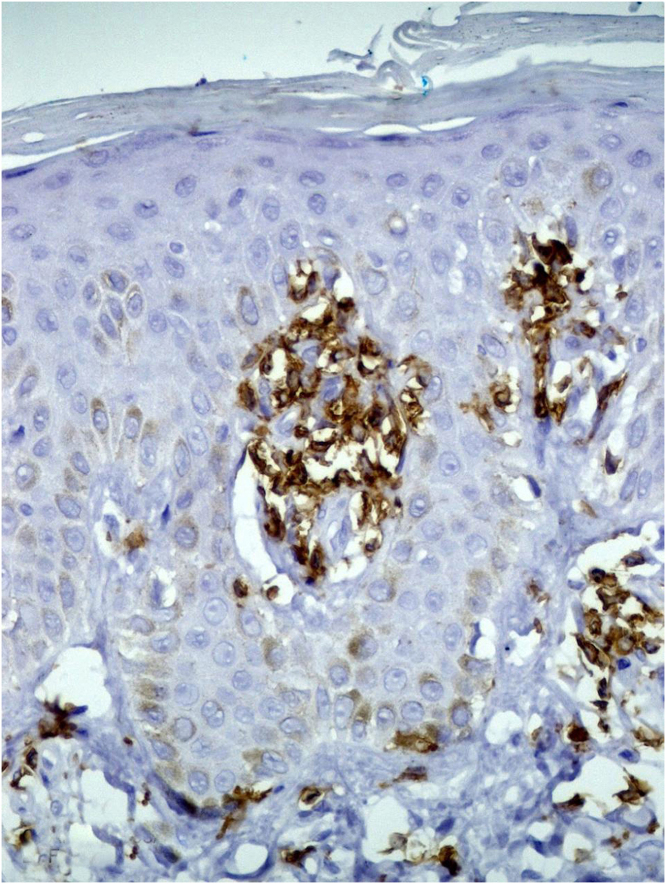
Hyperchromic plaques with ichthyosiform desquamation on the anterior trunk region.

A previously healthy 30-year-old female patient had presented pruritic lesions in the lower limbs that extended to the trunk and upper limbs for two years. She reported mild weight loss (<10% of baseline weight in 6 months), with no fever or night sweats. She denied using any medications. On physical examination, she had hyperchromic plaques with ichthyosiform desquamation on the trunk, axillae, abdomen, thighs and feet (Figure 1, Figure 2). The peripheral lymph nodes, liver and spleen were not palpable. No other changes were observed on physical examination.

Cervical, chest, abdominal, and pelvic CT scans showed no changes. Complete blood count, platelet levels, liver and kidney function, lactic dehydrogenase, beta-2-microglobulin, and TSH levels were within the normal limits; antinuclear factor (ANF) was 1/640, with nuclear coarse stippled or reticulated appearance; serology for cytomegalovirus and Epstein-Barr showed negative IgM and positive IgG; HTLV, HIV, viral hepatitis B and C serologies were non-reactive

**Figure 3:**
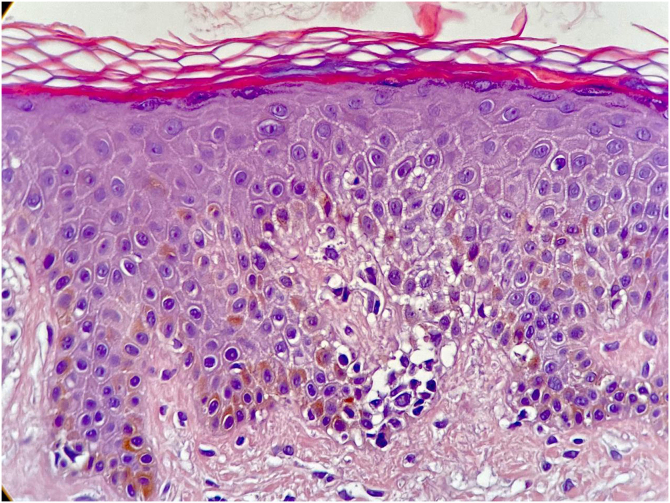
Orthokeratotic hyperkeratosis hypogranulosis and exocytosis of atypical lymphocytes (Hematoxylin & eosin, ×40).

**Figure 4:**
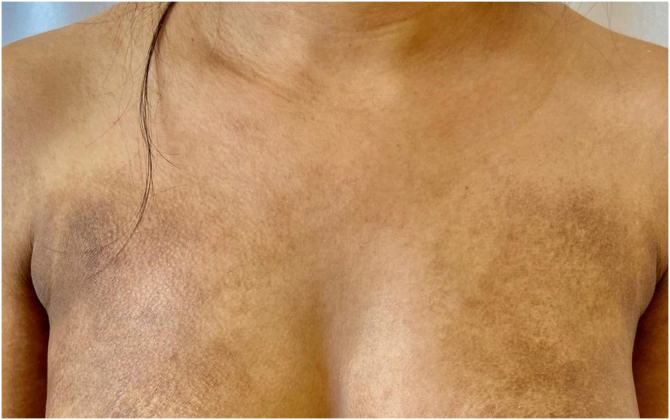
Immunohistochemistry showing CD5 positivity in epidermotropic atypical lymphocytes.

A skin biopsy showed hyperkeratosis with orthokeratosis, thinning of the granular layer, and exocytosis of atypical lymphocytes, grouped in Pautrier microabscesses. (Fig. 3). The lymphocytes were reactive with CD3, CD5 and CD8, but there was loss of CD7 reactivity (Fig. 4).

## What is your diagnosis?

  a) Ichthyosiform mycosis fungoides?
  b) Acquired ichthyosis associated with systemic malignancy (ovarian, thyroid, breast, lung cancer)?
  c) Ichthyosis associated with the use of medications (hydroxyurea, clofazimine, statins)?
  d) Ichthyosis associated with infectious disease (leprosy, AIDS, HTLV-1 infection)?

## Discussion

Ichthyoses can be hereditary or acquired. The hereditary forms are usually present at birth and their onset may occur later until the age of 13.^1^ Acquired ichthyoses (AI) can be associated with different etiologies, such as malignant, infectious, autoimmune diseases, endocrinological and nutritional disorders, kidney and liver failure, drug reactions and sarcoidosis. They may manifest before or after the identification of the systemic disease.^1^, ^2^

Mycosis fungoides (MF) is the most common form of primary cutaneous T-cell lymphoma and presents a variety of clinical manifestations.^3^ An ichthyosiform eruption as the only manifestation of MF is a rare event (1.8% of cases).^4^

Ichthyosiform mycosis fungoides (IMF) is characterized by an indolent course of the disease and good prognosis, with a mean age of onset at 32 years.^5^, ^6^ It may coexist with other subtypes of MF, especially the folliculotropic one, or manifest only as ichthyosiform lesions,^5^ with a clinical course similar to ichthyosis vulgaris or other forms of ichthyosis.^4^, ^5^ They are preferentially located on the trunk and extremities, but the entire body surface may be affected.^5^ Serology for HTLV is important to rule out the diagnosis of adult T-cell lymphoma/leukemia in these cases.

Although the clinical characteristics of IMF are indistinguishable from other causes of AI, histopathology discloses epidermotropic lymphocytic infiltrates, associated with typical characteristics of acquired ichthyosis such as orthokeratotic hyperkeratosis and thinning of the granular layer.^2^, ^3^ Immunohistochemical evaluation of IMF usually shows CD3+ and CD4+ lymphocytes but there have been reports of a predominance of CD8+ lymphocytes, as in the reported case.^4^, ^5^

Although ichthyosiform lesions can coexist with typical MF lesions, the diagnosis of IMF should be considered when ichthyosis is the sole manifestation of MF.^4^ Therefore, patients with acquired ichthyosis should be carefully evaluated to rule out this peculiar clinical-pathological variant.

## Financial support

None declared

## Authors’ contributions

Luciana Baptista Pereira: Conception and patient diagnosis, data collection, article writing, approval of the final version of the manuscript.

Natália de Paiva Sobreira: Conception of the article, data collection, writing of the article, approval of the final version of the manuscript.

Vanessa Barreto Rocha: Conception of the article, writing of the manuscript, approval and review of the final version of the manuscript.

## Conflicts of interest

None declared.
